# An Innovations-Based Noise Cancelling Technique on Inverse Kepstrum Whitening Filter and Adaptive FIR Filter in Beamforming Structure

**DOI:** 10.3390/s110706816

**Published:** 2011-06-29

**Authors:** Jinsoo Jeong

**Affiliations:** Faculty of Biomedical Engineering and Health Science, Universiti Teknologi Malaysia, 81310 UTM Skudai, Johor, Malaysia; E-Mails: jinsoojeong1015@hanmail.net; jeong@utm.my; Tel.: +60-7-553-5738; Fax: +60-7-553-5430

**Keywords:** innovations, whitening, ANC, beamforming, inverse kepstrum, adaptive FIR filter, system identification, acoustic transfer function

## Abstract

This paper presents an acoustic noise cancelling technique using an inverse kepstrum system as an innovations-based whitening application for an adaptive finite impulse response (FIR) filter in beamforming structure. The inverse kepstrum method uses an innovations-whitened form from one acoustic path transfer function between a reference microphone sensor and a noise source so that the rear-end reference signal will then be a whitened sequence to a cascaded adaptive FIR filter in the beamforming structure. By using an inverse kepstrum filter as a whitening filter with the use of a delay filter, the cascaded adaptive FIR filter estimates only the numerator of the polynomial part from the ratio of overall combined transfer functions. The test results have shown that the adaptive FIR filter is more effective in beamforming structure than an adaptive noise cancelling (ANC) structure in terms of signal distortion in the desired signal and noise reduction in noise with nonminimum phase components. In addition, the inverse kepstrum method shows almost the same convergence level in estimate of noise statistics with the use of a smaller amount of adaptive FIR filter weights than the kepstrum method, hence it could provide better computational simplicity in processing. Furthermore, the rear-end inverse kepstrum method in beamforming structure has shown less signal distortion in the desired signal than the front-end kepstrum method and the front-end inverse kepstrum method in beamforming structure.

## 1. Introduction

During the last five decades, noise cancelling and signal enhancing techniques have been developed. The techniques are fundamentally based on spectral subtraction, cepstrum and complex cepstrum methods using single-microphone sensor, and ANC and beamforming methods using multiple-microphone array sensors.

Since research on echo cancellation using adaptive filters and two-microphone sensors started in 1965, the adaptive filtering technique has been used as a solution tool for signal enhancing and noise cancelling schemes [1]. In 1975, Widrow *et al.* [2] proposed an FIR least mean square (LMS) algorithm-based ANC method using two microphones. This method requires that the primary microphone takes signal plus noise and the reference microphone takes noise alone, whereby the adaptive filter estimates noise statistics from the reference microphone and then uses them to minimize the output power in a minimizing mean square error (MMSE) calculation. From the theory, the application shows a practical problem due to the difficulty of separation between the period of the noise alone and the period of signal plus noise. This causes signal leakage into the reference microphone, which makes the adaptive filter estimate noise as well as the desired signal, hence results in limitations in the maximum cancellation from the output signal-to-noise ratio (SNR) with signal distortion.

Pulsipher *et al.* [3] have analyzed an unknown system used for the identification of acoustic path transfer functions between two-microphone sensors and noise sources from the ANC method and investigated the adaptive filter estimates of the ratio of acoustic path transfer functions for the correlated noise and found out that the problem comes from the nonminimum phase component of the ratio of acoustic transfer functions. To resolve it, the application uses a large amount of adaptive filter weights.

Harrison *et al.* [4] have introduced a new approach in the ANC method, whereby they use a noise estimating technique of a small separation between two-microphone sensors with the use of a voice activity detector (VAD) during the noise alone period. The result has shown that it could significantly reduce the amount of adaptive filter weights required for noise cancellation while minimizing the presence of reverberation. Nevertheless, the ANC method shows a limitation in the maximum cancellation in SNR because this maximum cancellation is related to the coherence level of the noise between two-microphone sensors [5], whereby it requires a significant coherence for even modest noise cancelling performance. The application of close and direct application of a desired signal [6–8] in front of two microphones with the use of adaptive filter using VAD during the noise alone period may reduce signal distortion.

The beamforming technique has been introduced to maximize signal directivity, therefore it increases the performance in SNR. This method may require many microphones with accurate phase alignment among the microphones array and hence, a computational complexity in processing is expected. To increase the performance in SNR, the technique has been developed with the use of adaptive filter and also VAD [9].

The cepstrum processing technique [10] may provide a solution for signal separation, but with the practical limitation due to minimum phase information. The complex cepstrum method [11] may give the solution for the minimum phase problem, where this minimum phase information can be directly estimated from the power spectrum.

The kepstrum [12,13] is similar to the complex cepstrum due to the fact that the minimum phase spectral factor can be directly obtained from a power spectrum estimation and it is equivalent with logarithmic minimum phase transfer function. The kepstrum method has been used as a system identification technique for unknown systems from the acoustic path transfer functions between microphone sensors input and the noise source in beamforming structure [14,15], with a phase recovering technique from the causal kepstrum domain, where the front-end kepstrum method has produced an improved SNR performance in a different input SNR for real-time processing in reverberant room environments. It has also been tested in different locations on a noise source using nonstationary music sound, tuned to a radio station. With a kepstrum method, a technique for preventing signal distortion has been used with the modified application [6–8] to a desired signal, and also modified application [4] to an adaptive filter with the use of VAD for differentiating the periods between signal plus noise and noise alone.

In addition, the kepstrum has a distinction in the case of signal plus noise, where the logarithmic minimum phase transfer function becomes the minimum phase kepstrum spectral factor and it can be represented as a Kolmogorov [16] power series expansion. Furthermore, the signal and noise may be implemented in an innovations-based form, where it was originally discovered by Kalman and Bucy [17] and it may then be applied to an infinite impulse response (IIR) Wiener filtering structure [18]. For the innovations-based approach, it may be represented as an output of normalized minimum phase spectral factor from innovations white noise input and has been used by Moir and Barrett [19].

By applying innovations-based whitening application in an ANC structure, it has been investigated in a simulation test, where it has been applied with the use of a FIR normalized least mean square (NLMS) algorithm for noise cancellation [20] and also with a FIR recursive least squares (RLS) algorithm [21], where it has been found that the application of innovations-based inverse kepstrum to cascaded adaptive filter gives a stable and causal system because all poles and zeros of the system are converted into unit circles due to the fact that the whitening application in the reference input works as all-pass filter so that it allows only one acoustic path transfer function to be considered as the unknown system.

For the real-time processing using adaptive RLS filters, the innovations-based whitening application has been applied as a front-end application in beamforming structure with rear-end zero-model FIR RLS filters and it was found that it gives better noise cancelling performance than a pole-zero model IIR RLS filter in an ANC structure [22].

From the previous studies [14,20–22] based on modified applications [4,6–8], it has been found that there are important features to be further investigated to verify the performance. In this paper, by considering: (1) signal distortion in a desired signal on the instant application of noise statistics in reverberant environment, (2) noise reduction on noise characteristics with nonminimum phase components and its consistency on its inverted acoustic path transfer function, and (3) the use of small amount of adaptive FIR filter weights for the real-time processing, hence for the fast convergence in estimate on noise statistics, it is analyzed in both ANC and beamforming structures. The inverse kepstrum method is then applied to the rear-end (of sum-and-subtract function) in beamforming structure to verify the performance by comparing signal waveforms in the time domain, spectra in the frequency domain as well as estimated coefficients arrays of inverse kepstrum and weights arrays of adaptive FIR filter and its pole-zero placements in noise statistics. Furthermore, the rear-end inverse kepstrum method is also compared with the front-end kepstrum method [14], which uses identification of two paths with the ratio of overall acoustic path transfer function, and also with front-end inverse kepstrum method using a whitening application [22].

## 2. Analysis of Innovations-Based Inverse Kepstrum

This section describes the analysis of cepstrum and complex cepstrum (kepstrum) with the relation of minimum phase kepstrum, and also for its whitening application (inverse kepstrum). It shows that the minimum phase kepstrum coefficients may be obtained from the logarithm of the minimum phase transfer function (Section 2.1) and also from the logarithm of the minimum phase spectral factor (Section 2.2). For the signal and noise, it shows that it can be represented as an output of normalized minimum phase spectral factor from innovations white noise input (Section 2.3). Based on this, it shows that logarithm of inverse minimum phase transfer function may be implemented as the innovations form of the normalized minimum phase kepstrum spectral factor for the whitening application (Section 2.4).

### 2.1. Analysis of the Minimum Phase Transfer Function

It is known that the causal transfer function can be expressed by Schwarz’s classical formula [23] as:

(1) $$H_{+}(z)=\frac{1}{2\pi }\int_{0}^{2\pi }H_{\text{R}}(\lambda )\left(\frac{1+e^{\text{j}\lambda }z^{-1}}{1-e^{\text{j}\lambda }z^{-1}}\right)d\lambda ,|z|<1$$

where *λ* as the integration variable and *z* = *re^jw^*.

This Equation gives the causal transfer function *H*_+_(*z*) whose real part on the unit circle is *H_R_*(*w*). Based on this, the phase information can be recovered by Hilbert’s transform relation. The logarithm of minimum phase transfer function log*H_M_*(*z*) can be written as:

(2) $$\text{log}H_{M}(z)=\frac{1}{2\pi }\int_{-\pi }^{\pi }\text{log}|H_{M}(\lambda )|\left(\frac{1+e^{j\lambda }z^{-1}}{1-e^{j\lambda }z^{-1}}\right)d\lambda ,\;\;\;|z|<1$$

where log *|H_M_*(*λ*)| is the magnitude part of minimum phase logarithmic transfer function.

This indicates that the minimum phase transfer function may be expressed in terms of Schwarz’s formula and hence the minimum phase information can be recovered from Hilbert’s transform relation.

By defining that *z* = *e^jw^*, it can be described as magnitude and phase term:

(3) $$\text{log}H_{M}(z)=\text{log}H_{M}(e^{\mathrm{jw}})=\text{log}|H_{M}(e^{\mathrm{jw}})|+j\text{arg}[\text{log}H_{M}(e^{\mathrm{jw}})]$$

For the *N* -point discrete form:

(4) $$\text{log}H_{M}\left(\frac{2\pi }{N}k\right)=\left[c_{0}+\sum_{n=1}^{N-1}\left(\frac{1}{2}c_{-n}e^{j\frac{2\pi }{N}\mathrm{kn}}+\frac{1}{2}c_{n}e^{-j\frac{2\pi }{N}\mathrm{kn}}\right)\right]+j\left[\sum_{n=1}^{N-1}-\left(\frac{1}{2}c_{-n}e^{j\frac{2\pi }{N}\mathrm{kn}}-\frac{1}{2}c_{n}e^{-j\frac{2\pi }{N}\mathrm{kn}}\right)\right]$$

From Equation (4), the magnitude of logarithmic minimum phase transfer function is:

(5) $$\text{log}\left|H_{M}(e^{j\frac{2\pi }{N}k})\right|=\left[c_{0}+\sum_{n=1}^{N-1}\left(\frac{1}{2}c_{-n}e^{j\frac{2\pi }{N}\mathrm{kn}}+\frac{1}{2}c_{n}e^{-j\frac{2\pi }{N}\mathrm{kn}}\right)\right]$$

and the phase of the logarithmic minimum phase transfer function is:

(6) $$\text{arg}\;\left[H_{M}{(e}^{j\frac{2\pi }{N}k})\right]=j\left[\sum_{n=1}^{N-1}-\left(\frac{1}{2}c_{-n}e^{j\frac{2\pi }{N}\mathrm{kn}}-\frac{1}{2}c_{n}e^{-j\frac{2\pi }{N}\mathrm{kn}}\right)\right]$$

This shows that Equation (5) shows an even cepstrum function, hence minimum phase kepstrum coefficients can be processed in the cepstrum domain by multiplying by two (2) in the positive time series, except for the first zeroth coefficient in the time series.

### 2.2. Analysis of Minimum Phase Spectral Factor

From the power spectral density Φ(*z*) it can be represented as causal spectral factor *H^+^*(*z*) and anticausal counterpart *H*^−^(*z*) as:

(7) $$\Phi (z)=H^{+}(z)H^{-}(z)=H^{+}(z)H^{+}(z^{-1})$$

Let *z* = *z*^−1^, then it follows that Φ(*z*^−1^) = *H*^+^(*z*^−1^)*H*^+^(*z*)

It follows that Φ(*z*) = Φ(*z*^−1^). By defining *Z* = *e^jw^*, we now have a logarithmic power spectrum as:

(8) Φ(w)=Φ(−w)

(9) $$\Phi (w)=|H^{+}(e^{\mathrm{jw}}){|}^{2}$$

(10) $$\text{log}\;\Phi (w)=2\;\text{log}|H^{+}(e^{\mathrm{jw}})|$$

For the *N* -point discrete form:

(11) $$K\left(\frac{2\pi }{N}k\right)=\text{log}\;\Phi \left(\frac{2\pi }{N}k\right)=2\;\text{log}\left|H^{+}(e^{j\frac{2\pi }{N}k})\right|=2\left[k_{0}+\sum_{n=1}^{N-1}\left(\frac{1}{2}k_{-n}e^{j\frac{2\pi }{N}\mathrm{kn}}+\frac{1}{2}k_{n}e^{-j\frac{2\pi }{N}\mathrm{kn}}\right)\right]$$

As a result of the symmetry property of *k_n_*, it can be expressed as:

(12) $$K^{+}\left(\frac{2\pi }{N}k\right)=\left[2k_{0}+\sum_{n=1}^{N-1}\left(k_{n}e^{-j\frac{2\pi }{N}\mathrm{kn}}\right)\right]$$

Furthermore, since *k_n_* are real, only a half portion in length can be considered as:

(13) $$K^{+}\left(\frac{2\pi }{N}k\right)=2k_{0}+\sum_{n=0}^{(N/2)-1}k_{n}e^{-j\frac{2\pi }{N}\mathrm{kn}}$$

From Equation (13), it shows that the kepstrum coefficients can be processed in the causal kepstrum domain by halving the first zeroth coefficient with the remaining coefficients truncated in size to (*N* / 2) −1. By truncating in size less than (*N* / 2) −1, kepstrum now becomes complex cepstrum, which is an approximation of the theoretical mathematical construct [19].

### 2.3. Analysis of Minimum Phase Kepstrum Spectral Factor on Signal and Noise

**Figure 1.**
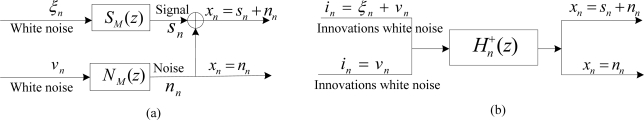
Equivalence of outputs based on inputs of: **(a)** white noise model and **(b)** innovations white noise model [upper part: signal plus noise, lower part: noise alone].

In the case of random signal plus noise, it can also be represented as innovations-based form. From Figure 1(upper part), it shows an equivalent relation of output from the inputs between white noise and innovations white noise as Equation (14):

(14) $$\Phi_{\mathrm{xx}}(z)=\Phi_{\mathrm{ww}}(z)+\Phi_{\mathrm{ss}}(z)=N_{M}(z)N_{M}(z^{-1})\sigma_{v}^{2}+S_{M}(z)S_{M}(z^{-1})\sigma_{\xi }^{2}=H_{n}^{+}(z)H_{n}^{-}(z)\sigma_{i}^{2}$$

where $\sigma_{v}^{2}$, $\sigma_{\xi }^{2}$ and $\sigma_{i}^{2}$ are the variances of the additive white noise, white noise input and white noise innovations process, respectively. *N_M_*(*z*) and *S_M_*(*z*) are coloured minimum phase transfer functions and *N_M_*(*z*^−1^) and *S_M_*(*z*^−1^) are maximum phase counterparts. $H_{n}^{+}(z)$ is the normalized minimum phase spectral factor, which has all its zeros inside |*z*| = 1 whilst $H_{n}^{-}(z)$ is the counterpart, which has its zeros outside |*z*| = 1.

In the case of signal plus noise, the logarithm of each positive- and negative-sided transfer function becomes the kepstrum spectral factors of the z- transform spectral density and these are represented as a power series expansion. For the innovations-based inverse kepstrum approach, signal plus noise are represented as an output of normalized minimum phase spectral factor from the innovations white noise input. It may be applied to an optimum IIR Wiener filtering structure, where it has been defined by Kailath [18] as combination of two cascaded filters, a front-end whitening filter to generate the white innovations process and cascaded spectral shaping filter to provide spectral shaping function for the input signal.

### 2.4. Analysis of Innovations-Based Inverse Kepstrum and its Application to Noise Signal Only

**Figure 2.**
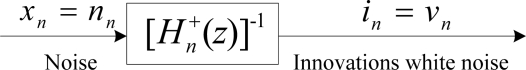
Representation of noise as innovations-based whitening form.

For the application of noise alone, it can be estimated during the absence of desired signal (Figure 1(lower part)). Therefore, the additive noise now can be represented as innovations-based form, as shown in Figure 2.

From the minimum phase kepstrum spectral factor of Equation (15):

(15) $$K^{+}(z)=\text{log}H^{+}(z)=k_{0}+k_{1}z^{-1}+k_{2}z^{-2}+\ldots \ldots$$

its exponentiation becomes a causal spectral factor as:

(16) $$H^{+}(z)=\text{exp}[K^{+}(z)]=\text{exp}(k_{0}+k_{1}z^{-1}+k_{2}z^{-2}+\ldots )$$

By using the fact that minimum phase spectral factor allows its invertibility, it can be expressed as:

(17) $${\left[H^{+}(z)\right]}^{-1}=\text{exp}[-K^{+}(z)]=\text{exp}[-(k_{0}+k_{1}z^{-1}+k_{2}z^{-2}+\ldots )]$$

This shows that the inverse of the minimum phase spectral factor can be obtained from the kepstrum exponential by multiplying by minus one (−1). It can also be represented in a normalized form:

(18) $$H^{+}(z)=\text{exp}[k_{0}]\text{exp}[k_{n}^{+}(z)]=\text{exp}[k_{0}]H_{n}^{+}(z)$$

(19) $$\text{log}H^{+}(z)=k_{0}+\text{log}H_{n}^{+}(z)$$

In the case of the application of additive noise alone, *i_n_* becomes *v_n_*. From the fact that *x_n_* = *H_M_*(*z*)*v_n_* and $x_{n}=H_{n}^{+}(z)\;i_{n}$, the relationship between minimum phase transfer function *H_M_*(*z*) and normalized minimum phase spectral factor $H_{n}^{+}(z)$ is described as:

(20) $$H_{M}(z)=H_{n}^{+}(z)\;i_{n}=H_{n}^{+}(z)v_{n}=H_{n}^{+}(z)\sigma_{ɛ}$$

where *v_n_*, *i_n_* and *σ_ɛ_* are white noise, innovations process and standard deviation of normalized innovations sequence respectively.

By taking logarithm of Equation (20):

(21) $$\text{log}H_{M}(z)=\text{log}H_{n}^{+}(z)+\text{log}\sigma_{ɛ}$$

From Equations (19) and (21) and with the fact that log *H*^+^(*z*) = log *H_M_*(*z*):

(22) $$k_{0}=\text{log}\sigma_{ɛ}$$

(23) $$\sigma_{ɛ}=\text{exp}[k_{0}]\;\text{and}\;\sigma_{ɛ}^{2}=\text{exp}[2k_{0}]$$

where *σ_ɛ_* and $\sigma_{ɛ}^{2}$ are standard deviation and variance of normalized innovations sequence respectively.

A normalized innovations-based inverse kepstrum is then represented from logarithmic minimum phase transfer function or logarithmic minimum phase spectral factor, which described as:

(24) $$\text{log}{\left[H_{M}(z)\right]}^{-1}=\text{log}{\left[H_{n}^{+}(z)\right]}^{-1}-\text{log}\sigma_{ɛ}=-K_{n}^{+}(z)-\text{log}\sigma_{ɛ}$$

Therefore, it shows that logarithm of inverse minimum phase transfer function may be implemented as innovations form of normalized minimum phase kepstrum spectral factor ( $\text{log}{\left[H_{M}(z)\right]}^{-1}=-K_{n}^{+}(z)$, assuming that standard deviation *σ_ɛ_* = 1).

## 3. Inverse Kepstrum Processing and Adaptive FIR NLMS Algorithm

**Figure 3.**
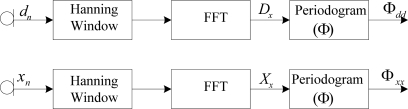
Periodogram estimate from each input microphone sensors.

For the whitening application, the inverse kepstrum is processed from the reference microphone *x_n_*. Therefore only the estimate from a single acoustic path transfer function is required. This may be compared with kepstrum processing, where the estimate of two acoustic path transfer functions from both the primary microphone *d_n_* and reference microphone *x_n_* is required. From each input microphone sensor, periodograms are obtained from Hanning-windowed fast Fourier transforms (FFTs) from the two-microphone inputs as shown in Figure 3.

As a discrete estimate of the continuous power spectral density, it uses a modified weighted overlapped segment averaging (WOSA) algorithm and the auto-periodograms are processed from 50% overlapping Hanning-windowed FFTs in 2,048 frame size by using smoothing method (25), with the forgetting factor *β* = 0.8 [20]:

(25) $$\Phi_{\mathrm{dd}}(i)=\beta \Phi_{\mathrm{dd}}(i-1)+(1-\beta )X_{d}(i)X_{d}^{*}(i)\;\;\;\Phi_{\mathrm{xx}}(i)=\beta \Phi_{\mathrm{xx}}(i-1)+(1-\beta )X_{x}(i)X_{x}^{*}(i)$$

**Figure 4.**
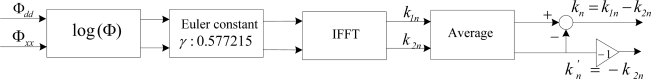
Block diagram for the comparison between inverse kepstrum processing (−*k*_2n_) and kepstrum processing (*k*_1n_ − *k*_2n_).

From (25), its logarithm needs to add Euler’s constant to be unbiased due to bias in magnitude [24]. Hence, each kepstrum coefficient from the two-microphone inputs are found from the inverse FFT (IFFT) of the unbiased logarithmic auto-periodograms and then by subtracting two kepstrum coefficient vectors (*k*_1n_ − *k*_2n_), we can get the kepstrum coefficients (*k*_n_) for kepstrum processing. On the other hand, inverse kepstrum coefficients ( $k_{n}^{'}$) are found by negating the kepstrum coefficients (− *k*_2n_) from the reference microphone. The processing difference between inverse kepstrum method and kepstrum method can be found in Figure 4.

This indicates that inverse kepstrum processing requires only a negative sign of the kepstrum coefficients, which can be obtained for the inverse of acoustic path transfer function from the reference microphone input Equation (26). The negated kepstrum coefficients are then normalized by dividing the zeroth kepstrum coefficient value:

(26) $$\text{log}\{1/H_{2}(z)\}↔K^{\prime }(z)=-K_{2}(z)$$

On the other hand, it is compared with the kepstrum method, which uses the ratio of the acoustic transfer function between two-microphone inputs as Equation (27).

(27) $$\text{log}\{H_{1}(z)/H_{2}(z)\}↔K(z)=K_{1}(z)-K_{2}(z)$$

**Figure 5.**
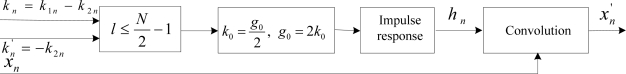
The conversion procedure from inverse kepstrum ( $k_{n}^{'}$) or kepstrum (*k*_n_) to impulse response (*h_n_*), and its convolution with reference input signal (*x_n_*).

Both the kepstrum and inverse kepstrum coefficients are transformed into the corresponding impulse response using recursive formula [12] and then it is convolved with reference input signal to get a refined new input signal as shown in Figure 5.

For the cascaded adaptive FIR filter, the NLMS algorithm [25] has been used and the weights are updated as:

(28) $$w_{n+1}=w_{n}+\frac{\mu X_{n}e_{n}}{0.0001+{\left\|X_{n}\right\|}^{2}}$$

where *μ* is step size (0 < *μ<* 2), ||*X_n_*||^2^ is input power and the value of 0.0001 is used to prevent zero division.

For the comparison of processing among: (i) inverse kepstrum coefficients, (ii) kepstrum coefficients and (iii) NLMS algorithm based adaptive FIR filter weights, computational complexity in floating point operations per second (FLOPS) can be compared as shown in Table 1, where it shows that inverse kepstrum processing gives the least computational complexity, and kepstrum processing shows less computational complexity than the ordinary adaptive FIR NLMS algorithm [20].

## 4. Inverse Kepstrum Method

**Figure 6.**
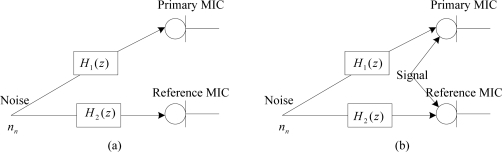
**(a)** Acoustic path transfer functions as noise estimate and **(b)** application of desired signal to the two microphones.

The inverse kepstrum method uses the whitening application from one acoustic path transfer function, *H*_2_(*z*) from the reference microphone during the noise alone period as shown in Figure 6(a), where the inverse kepstrum is to be analyzed as the front-end application to the adaptive FIR filter from the ANC structure, and the rear-end application to the sum-and-subtract function from the beamforming structure accordingly. It is also to be compared with the kepstrum method, which is based on identification of the ratio of acoustic path transfer functions, *H*_1_(*z*) and *H*_2_(*z*). During the signal and noise period, the noise estimate is applied to obtain the desired signal with a non-distortion. For the purpose, the method uses direct application of desired signal in front of two microphones as shown in Figure 6(b). Direct application of the desired signal can be found in the ANC structure [6] and also in beamforming structure [7,8].

### 4.1. Inverse Kepstrum Method as Front-End Application to ANC Structure

**Figure 7.**
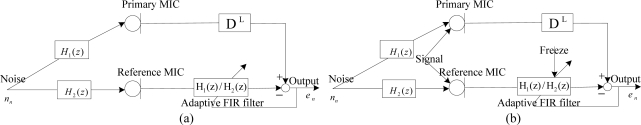
Analysis of identification of acoustic path transfer functions by an adaptive FIR filter in the ANC structure during the periods of **(a)** noise alone and **(b)** signal plus noise.

In the ANC structure, identification as an unknown system is represented as the ratio of acoustic transfer functions, *H*(*z*) = *H*_1_(*z*)/*H*_2_(*z*) as shown in Figure 7, where an ordinary adaptive FIR filter may be used to estimate the noise statistics during noise alone period. Its estimate is then applied to the signal and noise period to cancel the noise by using the estimated noise statistics with the condition that the desired signal should be only retained with no distortion.

**Figure 8.**
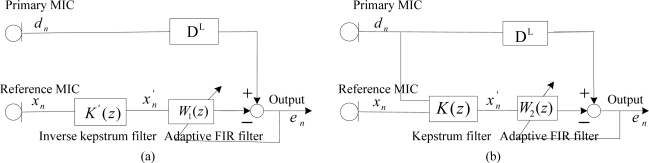
Application of **(a)** inverse kepstrum filter and **(b)** kepstrum filter to the ANC structure.

Secondly, the inverse kepstrum is applied in front of the adaptive FIR filter as shown in Figure 8(a), where the inverse kepstrum filter estimates a denominator part, 1/*H*_2_(*z*) and the cascaded adaptive FIR filter estimates a numerator part, *H*_1_(*z*) from the ratio of overall transfer function. It is compared with the kepstrum method as shown in Figure 8(b), where the kepstrum filter estimates the minimum phase term only from the ratio of overall transfer function and the cascaded adaptive FIR filter estimates remaining term and hence it works as an all-pass filter.

**Figure 9.**
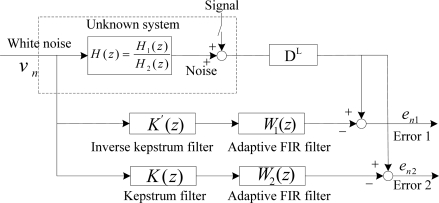
Block diagram for the operation comparison between an inverse kepstrum filter and a kepstrum filter as front-end application to an ANC structure.

Assuming that each acoustic path transfer functions between two microphones and noise source are as given in (29), it is represented as the unknown system of (30). Its estimates are to be analyzed as shown in Figure 9, where the operation of the inverse kepstrum is to be compared with the kepstrum filter as a front-end application to the cascaded adaptive FIR filter.

As a simple example, we assume that one transfer function from acoustic path transfer functions is nonminimum phase term, such as:

(29) $$H_{1}(z)=1+2z^{-1}\;\;\text{and }H_{2}(z)=1+0.2z^{-1}$$

where *H*_1_(*z*) is nonminimum phase transfer function.

The unknown system is then described as the ratio of transfer functions and it is represented as:

(30) $$H(z)=(1+2z^{-1})/(1+0.2z^{-1})=1+1.8z^{-1}-0.36z^{-2}+0.072z^{-3}-0.0144z^{-4}\ldots \ldots$$

where it can be estimated by ordinary adaptive FIR filter.

For the operation of the inverse kepstrum filter *K*′(*z*) and adaptive FIR filter *W*_1_(*z*), each one is estimated as:

(31) $$K^{\prime }(z)=1/(1+0.2z^{-1})\;\;W_{1}(z)=1+2z^{-1}$$

This indicates that the front-end inverse kepstrum *K*′(*z*) estimates the denominator part and the cascaded adaptive FIR filter *W*_1_(*z*) estimates the numerator part from the ratio of overall transfer functions. On the other hand, for the operation of the kepstrum filter *K*(*z*) and adaptive FIR filter *W*_2_(*z*), each one is estimated as:

(32) $$K(z)=(1+0.5z^{-1})/(1+0.2z^{-1})\;\;W_{2}(z)=(1+2z^{-1})/(1+0.5z^{-1})$$

This indicates that the front-end kepstrum estimates the minimum phase term only from the ratio of overall transfer function, where nonminimum phase term is reflected to the minimum phase term by a reciprocal polynomial as *z^−n^ H*(*z*^−1^). The cascaded adaptive FIR filter works then as an all-pass filter. From (31) and (32), we now have found that both methods show the same result as (30).

Now let us check with inverse of overall transfer function, such that:

(33) $$H_{1}(z)=1+0.2z^{-1}\;\text{and }H_{2}(z)=1+2z^{-1}$$

where *H*_2_(*z*) is nonminimum phase transfer function.

This indicates that the transfer function now has a nonminimum phase term in the denominator polynomial from the ratio of overall transfer functions.

The unknown system can be estimated from the ratio of transfer functions and it is represented as:

(34) $$\begin{array}{ll} H(z)= & \left(1+0.2z^{-1})/(1+2z^{-1})=[(1+0.2z^{-1})/(1+0.5z^{-1})][(1+0.5z^{-1})/(1+2z^{-1})\right]\\ & =(1+0.2z^{-1})/(1+0.5z^{-1})=1-0.3z^{-1}+0.15z^{-2}+\ldots \ldots \end{array}$$

where it can also be estimated by adaptive FIR filter. The Equation (34) indicates that the nonminimum phase term of denominator part is converted to a minimum phase term for the operation of an adaptive FIR filter so that the cascaded remaining part of Equation (34) always works as an all-pass filter.

The inverse kepstrum and cascaded adaptive FIR filter are estimated as:

(35) $$K^{\prime }(z)=1/(1+0.5z^{-1})\;\;W_{1}(z)=1+0.2z^{-1}$$

and the kepstrum and cascaded adaptive FIR filter are estimated as:

(36) $$K(z)=(1+0.2z^{-1})/(1+0.5z^{-1})\;\;\;W_{2}(z)=1$$

This indicates that the estimates, Equations (35) and (36), from both methods are the same as Equation (34), which is the inverse of the overall transfer function. The nonminimum phase component from the polynomial numerator and denominator of the overall transfer function may frequently occur in reverberant environments [26] and causes a fluctuation in the spectrum due to the difference between Equations (30) and (34). To deal with this problem, it indicates that neither the kepstrum nor the inverse kepstrum methods provide a solution in the ANC structure. Alternatively, we may solve this problem by swapping the position of the two microphones, but it is not a practical solution in a realistic environment.

### 4.2. Inverse Kepstrum Method as a Rear-End Application in Beamforming Structure

**Figure 10.**
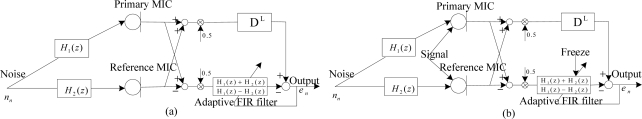
Analysis of identification of acoustic path transfer functions by adaptive FIR filter in beamforming structure during the periods of **(a)** noise alone and **(b)** signal plus noise.

In a beamforming structure, identification as an unknown system is represented as the ratio of combined acoustic transfer functions, *H*(*z*) = 0.5(*H*_1_(*z*) + *H*_2_(*z*))/0.5(*H*_1_(*z*) − *H*_2_(*z*)) as shown in Figure 10, where an ordinary adaptive FIR filter may also be used to estimate the ratio of combined overall transfer functions. Its estimate may then be applied to the signal and noise period to cancel the noise by using estimated noise statistics.

**Figure 11.**
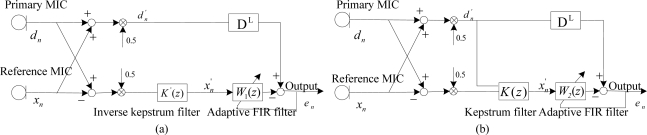
Rear-end application of **(a)** inverse kepstrum filter and **(b)** kepstrum filter to beamforming structure.

Based on this, an inverse kepstrum filter is applied in front of the adaptive filter as a rear-end application from the sum-and-subtract function from the beamforming structure as shown in Figure 11(a), where the inverse kepstrum filter estimates the polynomial denominator part, 1/0.5(*H*_1_(*z*) − *H*_2_(*z*)) and the cascaded adaptive FIR filter estimates the polynomial numerator part, 0.5(*H*_1_(*z*) + *H*_2_(*z*)) from the ratio of combined overall transfer functions. It is compared with the kepstrum method as shown in Figure 11(b), where the kepstrum filter estimates the minimum phase term only from the numerator polynomial part, 0.5(*H*_1_(*z*) + *H*_2_(*z*)) and the cascaded adaptive FIR filter estimates the remaining part from the numerator polynomial part, 0.5(*H*_1_(*z*) + *H*_2_(*z*)), where this numerator polynomial part is to be an overall transfer function with a delay filter in the rear-end primary input, $d_{n}^{'}$.

**Figure 12.**
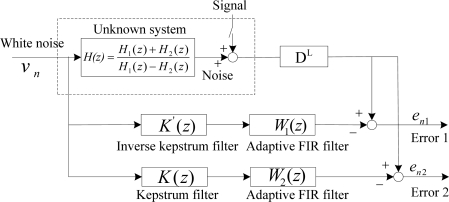
Block diagram for the operation comparison of an inverse kepstrum filter and a kepstrum filter as rear-end applications to a beamforming structure.

To compare with the operation in an ANC structure, the same components of the acoustic transfer functions are used as Equation (29) and this is represented as an unknown system as in Equation (37). It is to be analyzed as shown in Figure 12, where the operation of the inverse kepstrum is to be compared with the kepstrum filter as a rear-end application to the sum-and-subtract function of the beamforming structure.

From the unknown system, we have the numerator polynomial part from the ratio of overall combined transfer functions and it is represented as an overall transfer function and the denominator polynomial part works as a delay filter, described in Equation (37):

(37) $$H(z)=0.5[(1+2z^{-1})+(1+0.2z^{-1})]=1+1.1{\text{z}}^{-1}\;\;\text{with one sample delay},\;D^{-1}$$

For the operation of inverse kepstrum filter *K*′(*z*) and adaptive FIR filter *W*_1_(*z*), each one is estimated as:

(38) $$K^{\prime }(z)=1\;\text{and}\;W_{1}(z)=1+1.1z^{-1}\;\text{with one sample delay},\;D^{-1}$$

This indicates that the rear-end inverse kepstrum *K*′(*z*) works as a whitening filter with one sample delay and a cascaded adaptive FIR filter *W*_1_(*z*) estimated a numerator part from the ratio of combined overall transfer functions. On the other hand, for the operation of the kepstrum filter *K*(*z*) and the adaptive FIR filter *W*_2_(*z*), each one is estimated as:

(39) $$K(z)=1+0.9z^{-1}\;\text{and}\;W_{2}(z)=1+0.2z^{-1}\;\text{with one sample delay},\;D^{-1}$$

This indicates that the rear-end kepstrum estimates the minimum phase term only from the polynomial numerator part from the ratio of combined overall transfer functions, where the nonminimum phase term is reflected to the minimum phase term by the reciprocal polynomial as *z^−n^ H*(*z*^−1^). The cascaded adaptive FIR filter estimates the remaining term from the polynomial numerator of the ratio of combined overall transfer functions. Based on this, the unknown system Equation (37) may be estimated by the operations (38) and (39) as rear-end applications of the inverse kepstrum and kepstrum methods to the sum-and-subtract function of the beamforming structure, respectively.

Let us now check with the inverse of the overall transfer function, (33), where nonminimum phase term in denominator polynomial is no longer exist and overall transfer function is now obtained from numerator polynomial part of the ratio of overall combined transfer function.

Inverse kepstrum and cascaded adaptive FIR filter estimates as:

(40) $$K^{\prime }(z)=1\;\text{and }W_{1}(z)=1+1.1z^{-1}\;\text{with one sample delay},\;D^{-1}$$

where inverse kepstrum filter works as whitening filter and adaptive FIR filter estimates numerator polynomial part of the ratio of overall combined transfer function.

On the other hand, kepstrum and cascaded adaptive FIR filter estimates as:

(41) $$K(z)=1+0.9z^{-1}\;\text{and }W_{2}(z)=1+0.2z^{-1}\;\text{with one sample delay},\;D^{-1}$$

where kepstrum filter works as minimum phase filter and adaptive FIR filter estimate remaining term from the numerator polynomial part of the ratio of overall combined transfer function.

It shows that the estimates, Equations (40) and (41) by both methods on the inverted transfer function are same as Equations (38) and (39), which are the estimates by the both methods on the direct transfer function. It indicates that both kepstrum and inverse kepstrum methods do provide a solution in beamforming structure, which may give a practical solution in a reverberant noise with nonminimum phase component from overall transfer function because it does not need to swap the two microphones position. The detailed analysis on nonminimum phase transfer function has been investigated between ANC and beamforming structures [27].

## 5. Experiments

Experiments were implemented in both simulation tests on pc software and real tests using real nonstationary noise in a room environment. According to the main three considerations (signal distortion in the desired signal, noise reduction performance in noise with nonminimum phase components and convergence level in estimates of noise statistics with the use of a small amount of adaptive FIR filter weights), the performances achieved when using an inverse kepstrum filter were verified in both the ANC and beamforming structures. Furthermore, the rear-end application of the inverse kepstrum method in beamforming structure was also compared with two front-end applications of the kepstrum method [14] and the inverse kepstrum method [22] in beamforming structure.

The methodology is based on the fact that the coefficients (kepstrum and inverse kepstrum) and weights (adaptive FIR filter) are continuously updated during noise alone period to estimate noise statistics. When the desired signal is applied to noise, the last updated coefficients and weights are frozen and applied to the desired signal and noise. For a precise test, it is programmed to stop updating coefficients and weights, and then these are applied to the desired signal and noise period. To check the strength in amplitude and distortion status in desired signal, simple three sine waveforms are added and used as the desired signal for both simulation and real tests. For the test using real noise, we use a nonstationary music sound, tuned to a certain radio station. For the use of an adaptive FIR filter, an NLMS algorithm has been used with the use of step size *μ* = 0.001 for the simulation test and *μ* = 0.5 for the real test. For the processing, 2,048 frame size, sampling frequency of 22,050 Hz and Nyquist frequency of around 11,000 Hz have been chosen. Two preamplifiers and two microphones of unidirectional electret condenser type are used, placed 7 cm distance apart in broadside configuration for the real test in a room [3.8 m(d) × 3 m(w) × 2.8 m(h)]. The performance is to be verified by comparing signal waveforms in time domain, spectra in frequency domain, estimated coefficients and weights arrays and its pole-zero placements.

### 5.1. Simulation Test

For the simulation test, the acoustic transfer functions of (29) are used as the unknown system, which has nonminimum phase component in noise. The desired signal, consisting of three frequencies (500 Hz, 550 Hz and 700 Hz), has arbitrarily been used as shown in Figure 14(a).

#### 5.1.1. Adaptive FIR filter in ANC and beamforming structures

The first test is to verify the noise cancelling performance in the ANC structure by applying three adaptive FIR filter weights for the noise characteristic with nonminimum phase component in the polynomial numerator (29) and nonminimum phase component in the polynomial denominator (33) in the acoustic transfer function. It is also verified in the beamforming structure.

**Figure 13.**
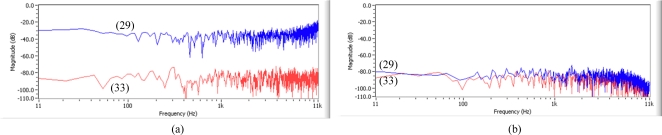
Comparison in spectra between direct transfer function (29) and its inverse transfer function (33) on application of adaptive FIR filter in **(a)** ANC structure and **(b)** beamforming structure.

From the simulation test based on the block diagram (Figure 9) of the ANC structure [Figure 7(a)], it is found that the noise spectrum with nonminimum phase term in the polynomial denominator (33) shows worse and much different performance than one of nonminimum phase term in the numerator (29), as shown in Figure 13(a). On the other hand, from the simulation test based on the block diagram (Figure 12) of the beamforming structure [Figure 10(a)], spectra of both Equations (29) and (33) show good and almost same spectral performance, as shown in Figure 13(b). From the test result, it is shown that the adaptive FIR filter works well with the use of a small amount of weights and shows consistency in spectra in the beamforming structure for the noise with both nonminimum phase cases, Equations (29) and (33). On the other hand, based on the test result in Figure 13(a), a fluctuation in spectrum is expected in ANC structure in the case that noise statistics is frequently changed in a reverberant environment.

**Figure 14.**
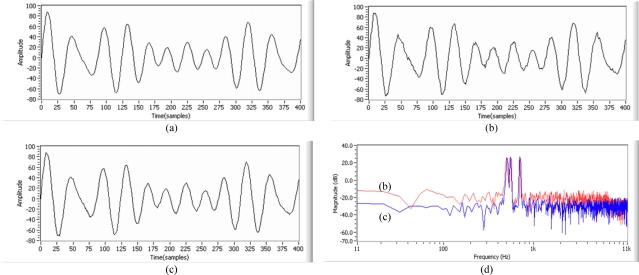
Simulation test based on 3 adaptive FIR filter weights: **(a)** waveform of original desired signal; **(b)** waveform of desired signal with three samples delayed in the ANC structure; **(c)** waveform of desired signal with no sample delay in the beamforming structure; **(d)** corresponding spectra of (b) and (c).

For the second test, the acoustic transfer function Equation (29) is estimated by using three adaptive filter weights and then it is applied to signal and noise. To get the best result, it is found that it is needed to set the three sample delay as D^−3^ to reduce signal distortion in the ANC structure. On the other hand, it is found that there is no need to set the delay in the beamforming structure. From the test result, by using a small amount of three adaptive FIR filter weights, it is shown that the performance in the beamforming structure provides no signal distortion in the desired signal without any delay adjustment and also better noise reduction in noise with nonminimum phase term than the performance in the ANC structure, as shown in Figure 14.

From the above two test results in terms of noise reduction performance in reverberant noise with nonminimum phase (Figure 13) and signal distortion in the desired signal (Figure 14), it is found that an adaptive FIR filter works better in a beamforming structure than in an ANC structure.

#### 5.1.2. Application of inverse kepstrum method to two structures and comparison with kepstrum

**Figure 15.**
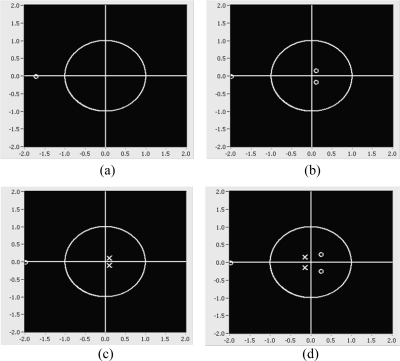
Snapshot of pole-zero placement: **(a)** one zero from adaptive FIR filter **(b)** increased to three zeros from (a) **(c)** two poles from inverse kepstrum coefficients and one zero from the adaptive FIR filter **(d)** two poles from kepstrum coefficients and increase to three zeros from the adaptive FIR filter.

The objective is to verify the performance of the inverse kepstrum method in ANC and beamforming structures. For the test in the ANC structure, the acoustic transfer functions of (29) have been used, hence the unknown system is expected to be estimated as (30). As verified in Table 2 (a)–(iii) and Figure 15 (a), an adaptive FIR filter using one zero does not approximate to (30). It can be approximated by increasing the adaptive FIR filter weights to four from (a) so that it gives almost the same performance as Equation (30), as shown in Table 2 (b)–(iii) and Figure 15(b). On the other hand, by applying two inverse kepstrum coefficients to a cascaded adaptive FIR filter using one zero, it also gives almost the same performance as Equation (30) as shown in Table 2 (c)–(iii) and Figure 15(c). For the comparison, with the use of same size of two kepstrum coefficients, it indicates that four adaptive filter weights are needed to approximate (30) as shown in Table 2 (d)–(iii) and Figure 15(d).

From the test result, it is analyzed that in the ANC structure, the application of an inverse kepstrum filter works well with an adaptive FIR filter in terms of convergence with a smaller amount in adaptive filter weights, rather than when the adaptive FIR filter is used with application of a kepstrum filter From the test results between (c)–(ii) and (d)–(ii) in Table 2, it indicates that inverse kepstrum method needs only 50% of the adaptive FIR filter weights needed by the kepstrum method.

**Figure 16.**
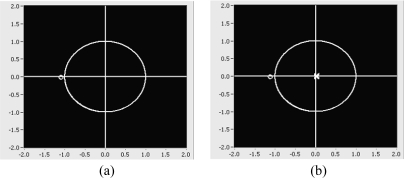

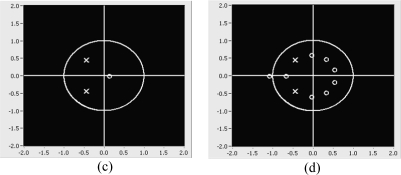
Snap shot of pole-zero placement: **(a)** one zero from adaptive FIR filter **(b)** two poles from inverse kepstrum and one zero from adaptive FIR filter **(c)** two poles from kepstrum filter and one zero from adaptive FIR filter **(d)** two poles from kepstrum filter and increased to eight zeros from adaptive FIR filter.

For the test in beamforming structure, acoustic transfer functions of Equation (29) has been used, hence unknown system is expected to estimate as Equation (37). As verified in Table 3 (a)–(iii) and Figure 16(a), adaptive filter using one zero is well approximated to Equation (37).

Based on this, by using two inverse kepstrum coefficients, it can be verified that it can also be approximated to Equation (37) as shown in Table 3 (b)–(iii) and Figure 16(b). On the other hand, by applying two kepstrum coefficients to the cascaded adaptive FIR filter using one zero, it cannot be approximated to (37), as shown in Table 3 (c)–(iii) and Figure 16(c). For the comparison, with the use of same size of two kepstrum coefficients, it indicates that eight adaptive filter weights are needed to approximate (37), as shown in Table 3 (d)–(iii) and Figure 16(d).

From the test result, it is also analyzed in a beamforming structure and shows that application of inverse kepstrum filter works well with the adaptive FIR filter in terms of convergence with much smaller amount in adaptive filter weights, rather than when the adaptive FIR filter is used with application of a kepstrum filter. From the test results between (b)–(ii) and (d)–(ii) in Table 3, it indicates that inverse kepstrum method needs only 25% (75% less) of the adaptive FIR filters than the kepstrum method.

From the comparison results between inverse kepstrum filter and kepstrum filter to adaptive FIR filter in terms of convergence in noise statistics and its pole-zero placements, it is found that the application of the inverse kepstrum filter could give a convergence benefit with the use of a small amount in adaptive FIR filter weights in the ANC structure [Table 2 (c)–(ii)], and is much more effective in a beamforming structure [Table 3 (b)–(ii)].

### 5.2. Real Tests

**Figure 17.**
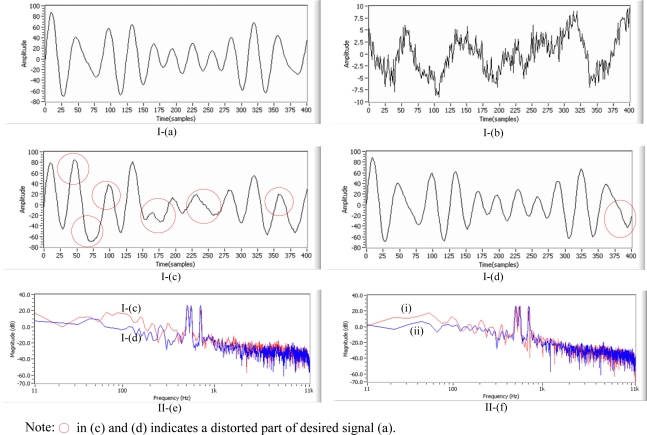
Real test in a room environment: (I) —waveforms of **(a)** desired signal **(b)** real nonstationary noise **(c)** processing with three samples delayed in ANC structure **(d)** processing with no delay in beamforming structure, (II) —corresponding spectra from **(e)** I-(c) and I-(d) **(f)** comparison between (i) processing with 200 adaptive FIR filter weights alone and (ii) processing with 32 inverse kepstrum coefficients and 50 adaptive FIR filter weights in beamforming structure.

The simulation test results suggest that the inverse kepstrum should achieve more noise reduction without signal distortion in the desired signal by using small amount of adaptive FIR filter for the real tests in a realistic reverberant environment. Therefore, the inverse kepstrum has been tested in a beamforming structure [Figure 11 I-(a)] in a room. To verify the performance in signal, the desired signal [Figure 17 I-(a)] consisting of three frequencies (500 Hz, 550 Hz and 700 Hz) has been used, and music sounds tuned to a radio station [Figure 17 I-(b)] has been used as real nonstationary noise in a room. From the test on the ANC structure, it is shown that the shape of the signal waveform has been distorted in time domain, as shown in Figure 17 I-(c). On the other hand, it is shown that almost the same shape of the signal waveform has been maintained in the beamforming structure as shown in Figures 17 I-(d). The performance has also been compared in spectra in frequency domain as shown in Figure 17 II-(e). In the beamforming structure, the performance has been compared between 200 adaptive FIR filter weights alone [Figures 17 II-(f)-(i)] and 32 inverse kepstrum coefficients with 50 cascaded adaptive FIR filter weights [Figure 17 II-(f)-(ii)], where the test results show that inverse the application of the kepstrum with reduced adaptive FIR filter weights gives even better noise reduction performance while maintaining the original desired signal. It indicates that the application of the inverse kepstrum could provide a benefit of more computational simplicity than a kepstrum in processing as well as the ordinary adaptive FIR filter (Table 1). To reduce the computational complexity, the alternative method could be considered by decreasing the window size but the problem has been found with the widening sidelobe on the desired signal. By using the inverse kepstrum, it has been found that increasing to 4096 the window size is acceptable for the real-time processing with the narrowing sidelobe on the desired signal [21].

**Figure 18.**
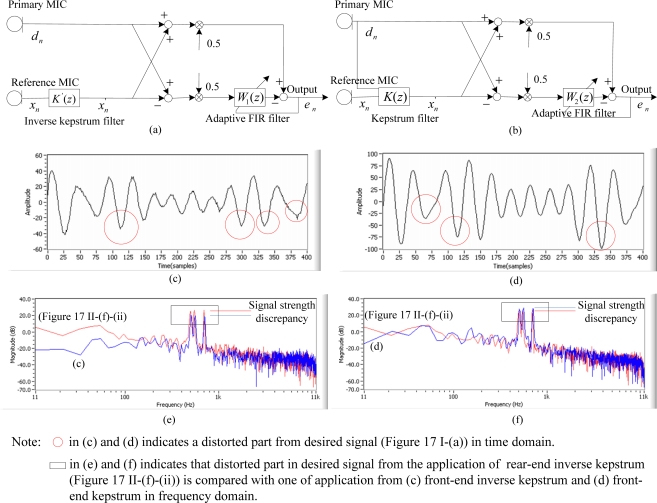
Front-end application of: **(a)** inverse kepstrum and **(b)** kepstrum in beamforming structure; **(c)** waveform in the time domain by inverse kepstrum; **(d)** waveform in time domain by kepstrum; **(e)** spectra in frequency domain between Figure 17 II-(f)-(ii) and (c); **(f)** its spectra in frequency domain between Figure 17 II-(f)-(ii) and (d); [commonly based on 32 inverse kepstrum coefficients (or 32 kepstrum coefficients) and 50 adaptive FIR filter weights].

Furthermore, the performance [Figure 17 II-(f)-(ii)] of the inverse kepstrum in rear-end beamforming [Figure 11(a)] has been compared with the front-end application of the inverse kepstrum [Figure 18(a)] and kepstrum [Figure 18(b) in a beamforming structure. With the use of a kepstrum (or inverse kepstrum) coefficients to 32 and a reduced size of adaptive filter weights to 50, it is now to verify the performance on signal distortion in a desired signal when the desired signal is applied to the noise estimates of coefficients and weights, which are abruptly frozen on the rapid change of noise statistics. To verify the performance, the average discrepancy between strengths of three frequencies after processing has been measured since each input frequency is applied with equal strengths in dB. For a trial test, the same desired signal is applied at random intervals to the frequently changing real noise environment, where the estimate of noise transfer function is randomly frozen accordingly. Figure 18 shows snapshot of a performance among the application of front-end kepstrum, front-end inverse kepstrum with comparison of application of rear-end inverse kepstrum in time and frequency domains.

As shown in Figure 18(c,e), the test results show that front-end inverse kepstrum provides better noise reduction but with attenuated amplitude in a desired signal. On the other hand, the front-end kepstrum [14] shows more strength in signal amplitude with almost same noise reduction as shown in Figure 18(d,f). However, it has been found from the test that both front-end methods [Figure 18(a,b)] are more vulnerable to signal distortion than the rear-end inverse kepstrum method in beamforming structure. As shown in Table 4, the level of signal distortion has been compared by measuring an average discrepancy of signal strength in dB from front-end kepstrum [14], front-end inverse kepstrum [22] and rear-end inverse kepstrum, where it has been calculated as 0.25 dB, 0.45 dB and 0.15 dB, respectively.

### 5.3. Summary

For real-time processing in a realistic reverberant environment, the kepstrum method and inverse kepstrum method have been applied to ANC and beamforming structures, hence its performance on modified application [4,6–8] to desired signal and adaptive filter has been investigated in detail in this paper.

Firstly, the reverberant nature of most rooms gives rise to nonminimum phase components in the acoustic transfer function [28] and its inverse is often required in a realistic reverberant environment [26], hence it might produce instability in processing. Therefore, the acoustic transfer function and its inverted transfer function have been tested in ANC and beamforming structures. Secondly, the acoustic noise transfer function changes rapidly and frequently in a realistic reverberant environment and the estimated noise statistics for the acoustic transfer function are abruptly frozen. It is then instantly applied during the desired signal period, which might cause a signal distortion. To verify the performance, a discrepancy in dB has been compared among the application of front-end kepstrum, front-end inverse kepstrum and rear-end inverse kepstrum in a beamforming structure. Thirdly, a fast convergence in adaptive filter application is essential in real-time processing so that a fast convergence with small amount of adaptive filter weights has been tested in both kepstrum method and inverse kepstrum method.

For an accurate discrepancy measurement in a desired signal, three added simple sine waveforms (disregarding whether it is narrowband or wideband in this test) have arbitrarily been used as a sample of a desired signal so that the distorted amount in a desired signal could be measured by calculating the average discrepancy in dB to check the consistency level of the signal strengths in dB from the original desired signal. For the precise testing in estimate of coefficients and weights, instead of using automatic VAD, it has been programmed to stop and make the last updated coefficients and weights to be frozen on demand of the application, and then it is applied to the desired signal and real noise period.

From the test results on main three considerations using the above mentioned methodology, it can be summarized that for the application of adaptive FIR filter to noise cancelling scheme, it is found that adaptive filter works better in a beamforming structure than the ANC structure in terms of signal distortion in desired signal, noise reduction in noise with nonminimum phase component (Figure 14) and consistency of estimate in noise statistics on its inverted transfer function (Figure 13).

For the rear-end application of the inverse kepstrum to beamforming structure, the inverse kepstrum method gives better convergence with a much smaller amount of adaptive FIR filter weights than the kepstrum method (Table 3), which contributes to better real-time processing (Table 1). It has also been found that the combination of rear-end inverse kepstrum and cascaded adaptive FIR filter gives better noise reduction performance with highly reduced adaptive filter weights than the sole application of an adaptive FIR filter (Figure 17). In addition, it gives less signal distortion in the desired signal than the front-end applications of kepstrum method and inverse kepstrum method (Figure 18, Table 4).

## 6. Conclusions

An adaptive FIR filter has shown a performance distinction between ANC and beamforming structures in terms of signal distortion in the desired signal and nonminimum phase in noise, where the beamforming structure provides better performance than the ANC structure on application of an adaptive FIR filter. Based on this, the innovations-based inverse kepstrum method has been applied to the beamforming structure, and its performance has also been compared with the kepstrum method and inverse kepstrum method as front-end applications in the beamforming structure. The simulation and real tests show that the innovations-based whitening inverse kepstrum method has provided a promising result in rear-end beamforming structure in terms of signal distortion in the desired signal, noise reduction in noise with nonminimum phase and convergence level in estimation of noise statistics with the use of a small amount of adaptive FIR filter weights. Furthermore, the inverse kepstrum method provides the computational simplicity in processing so that it could be a benefit to a practical real-time adaptive noise cancelling scheme. Further analysis and investigation of the inverse kepstrum method will be performed using ECG (electrocardiogram) signals in biomedical signal processing for noise cancelling schemes.

## Figures and Tables

**Table 1.** Comparison of computational complexity in FLOPS on (i) inverse kepstrum processing, (ii) kepstrum processing and (iii) NLMS algorithm [20].

**Algorithm**	**Required processing**	**FLOPS**
(i) Inverse kepstrum	1 × WOSA Periodogram 4 × FFT / IFFT 2 × Logarithm /Exponential	*IN* log_2_(5.12 / Δ*f*) 4(*N* / 2)log_2_*N* *2N*^1/3^(log *N*)*^2^*
	Total Computation (*)	0.057 × 10^6^
(ii) Kepstrum	2 × WOSA Periodogram 5 × FFT / IFFT 3 × Logarithm/Exponential	2*N* log_2_(5.12 / Δ*f*) 5(*N* / 2)log_2_*N* *3N*^1/3^(log *N*)^2^
	Total Computation (*)	0.08 × 10^6^
(iii) NLMS	Real multiplication (A) Iterations (B)	3*N*^2^ + 2*N* 20 × (A)
	Total Computation (**)	(A) 0.12 × 10^6^ (B) 2.4 × 10^6^

Note: Total computation:

(*) is based on N: 2048 frame size,

(**) is based on 200 NLMS weights.

**Table 2.** Weights and coefficients arrays showing each estimate output from the simulation test based on the block diagram in Figure 9.

(a) Adaptive filter weights (one zero)							
	1	1.712	–	–	–	–	–
(b) Adaptive filter weights (three zeroes)							
	1	1.800	−0.359	0.069	–	–	–
(c) (i) Inverse kepstrum coefficients (two poles) and (ii) Adaptive filter weights (one zeroes)							
(i)	1	−0.194	0.018	–	–	–	–
(ii)	1	1.992		–	–	–	–
(iii)	1	1.797	−0.369	0.037	–	–	–
(d) (i) Kepstrum coefficients (two poles) and (ii) Adaptive filter weights (three zeroes)							
(i)	1	0.299	0.044	–	–	–	–
(ii)	1	1.501	−0.846	0.234	–	–	–
(iii)	1	1.800	−0.352	0.048	0.032	0.010	–

Note: iii=i*ii, where * indicates convolution.

**Table 3.** Weights and coefficients arrays showing each estimate output from the simulation test based on the block diagram in Figure 12.

(a) Adaptive filter weights (one zero)							
	1	1.101	–	–	–	–	–
(b) (i) Inverse kepstrum coefficients (two poles) and (ii) Adaptive filter weights (one zero)							
(i)	1	0.004	0.000	–	–	–	–
(ii)	1	1.092	–	–	–	–	–
(iii)	1	1.097	0.005	0.000	–	–	–
(c) (i) Kepstrum coefficients (two poles) and (ii) Adaptive filter weights (one zero)							
(i)	1	0.953	0.045	–	–	–	–
(ii)	1	−0.150	–	–	–	–	–
(iii)	1	0.803	0.311	−0.068	–	–	–
(d) (i) Kepstrum coefficients (two poles) and (ii) Adaptive filter weights (eight zeroes)							
(i)	1	0.898	0.403	–	–	–	–
(ii)	1	0.200	−0.582	0.443	−0.163	−0.031	0.092
(iii)	1	1.098	−0.003	0.002	∼0.000	∼0.000	∼0.000

Note: (A) iii=i*ii, where * indicates convolution.

(B) (d) (ii) and (iii) shows only seven weights estimates in arrays.

**Table 4.** Comparison of average discrepancy on application of front-end kepstrum [14], front-end inverse kepstrum [22] and rear-end inverse kepstrum.

**Application**	**Front-end kepstrum**				**Front-end inverse kepstrum**				**Rear-end inverse kepstrum**			
Frequency (Hz)	500	550	700	D	500	550	700	D	500	550	700	D
Trial 1	30	29.5	29.5	0.25	20	20.5	21	0.5	25	25	24.5	0.25
Trial 2	29.5	30	30	0.25	21	20	20	0.5	25	25	25	0
Trial 3	29.5	30	29.5	0.25	20	20	20.5	0.25	25	25	25.5	0.25
Trial 4	30	29.5	30	0.25	21	20	21	0.5	25	25	25	0
Trial 5	29.5	29.5	30	0.25	21	20.5	20	0.5	25	24.5	25	0.25
Average D (dB)	0.25				0.45				0.15			

Note: D indicates discrepancy in dB from signal strength of desired signal in frequency domain.

## References

[1] Compernolle DV Switching Adaptive Filters for Enhancing Noisy and Reverberant Speech from Microphone Array Recordings.

[2] Widrow B, Glover JR, Mc Cool JM, Kaunitz J, Williams CS, Hearn RH, Zeidler JR, Dong E, Goodlin RC (1975). Adaptive noise cancelling: Principles and applications. Proc. IEEE.

[3] Pulsipher D, Boll SF, Rushforth C, Timothy L Reduction of Nonstationary Acoustic Noise in Speech Using LMS Adaptive Noise Cancelling.

[4] Harrison WA, Lim JS, Singer E (1986). A new application of adaptive noise cancellation. IEEE Trans. Acoust. Speech Signal Process.

[5] Goulding MM, Bird JS (1990). Speech enhancement for mobile telephony. IEEE Trans. Veh. Tech.

[6] Hussain A, Campbell DR, Moir TJ (1997). Multi-sensor sub-band adaptive noise cancellation for speech enhancement in an automobile environment. Adaptive Signal Processing for Mobile Communication Systems (Ref. No. 1997/383). IEE Colloquium.

[7] Greenberg JE, Zurek PM (1992). Evaluation of an adaptive beamforming method for hearing aid. J. Acoust. Soc. Am.

[8] Campbell DR, Shields PW (2003). Speech enhancement using sub-band adaptive Griffiths-Jim signal processing. Speech Commun.

[9] Griffiths LJ, Jim CW (1982). An alternative approach to linearly constrained adaptive beamforming. IEEE Trans. Antenna Propag.

[10] Bogert BP, Healy M, Turkey JW, Rosenblatt M (1963). The Frequency Analysis of Time Series for Echoes: Cepstrum, Pseudo-Autocovariance, Cross-Cepstrum and Saphe Cracking.

[11] Oppenheim AV, Schafer RW (1968). Homomorphic analysis of speech. IEEE Trans. Audio Electroacoust.

[12] Silvia MT, Robinson EA (1978). Use of the kepstrum in signal analysis. Geoexploration.

[13] Barrett JF, Moir TJ (1984). Spectrum analysis using kepstrum coefficients. IEE Colloq Recent Adv Ident Signal Process IEE Lond UK Part II.

[14] Jeong J, Moir TJ (2008). A real-time kepstrum approach to speech enhancement and noise cancellation. Neurocomputing.

[15] Jeong J (2007). A Kepstrum Approach to Real-Time Speech Enhancement.

[16] Kolmogorov AN (1941). Stationary sequences in Hilbert space. Bull. Math. Univ. Moscow.

[17] Kalman RE, Bucy RS (1961). New results in linear filtering and prediction theory. J. Basic Eng.

[18] Kailath T (1968). An innovations approach to least-squares estimation—part I: Linear filtering in additive white noise. IEEE Trans. Autom. Contr.

[19] Moir TJ, Barrett JF (2003). A kepstrum approach to filtering, smoothing and prediction with application to speech enhancement. Proc. R. Soc. Lond. A.

[20] Jeong J Analysis of Inverse Kepstrum and Innovations-Based Application to Noise Cancellation.

[21] Jeong J Inverse Kepstrum Approach to RLS Algorithm and Application to Adaptive Noise Cancelling.

[22] Jeong J, Lino GM (2011). Real-time noise cancelling approach on innovations-based whitening application to adaptive FIR RLS in beamforming structure. Adaptive Filtering.

[23] Schwarz HA (1872). Zur Integration der partiellen Differentiagleichung. J. Reine Angewandte Math.

[24] Wahba G (1980). Automatic smoothing of the log periodogram. J. Amer. Stat. Assoc.

[25] Haykin S (1996). Adaptive Filter Theory.

[26] Neely ST, Allen JB (1979). Invertibility of room impulse response. J. Acoust. Soc. Am.

[27] Jeong J Analysis of Nonminimum Phase Component on Direct Transfer Function and its Inverted Transfer Function.

[28] Lee B, Goudeseune C, Hasegawa-Johnson MA (2003). Open loop dereverberation of multichannel room impulse responses. J. Acoust. Soc. Am.

